# Quantitative MRI relaxometry in brain tumor needle biopsies: Multimodal comparison with tissue fluorescence, radiology, and neuropathology

**DOI:** 10.1371/journal.pone.0326765

**Published:** 2025-07-07

**Authors:** Elisabeth Klint, Anders Tisell, Ida Blystad, Martin Hallbeck, Teresa Nordin, Jan Hillman, Johan Richter, Karin Wårdell

**Affiliations:** 1 Department of Biomedical Engineering, Linköping University, Sweden; 2 Department of Radiation Physics in Linköping, and Department of Health, Medicine and Caring Sciences, Linköping University, Sweden; 3 Centre for Medical Image Science and Visualization (CMIV), Linköping University, Sweden; 4 Department of Radiology in Linköping and Department of Health, Medicine and Caring Sciences, Linköping University, Sweden; 5 Department of Clinical Pathology in Linköping, and Department of Biomedical and Clinical Sciences, Linköping University, Sweden; 6 Department of Neurosurgery in Linköping, and Department of Biomedical and Clinical Sciences, Linköping University, Sweden; 7 Department of Neurosurgery in Linköping, and Department of Biomedical Engineering, Linköping University, Sweden; Indiana University, UNITED STATES OF AMERICA

## Abstract

**Background:**

Quantitative MRI (qMRI) relaxometry holds potential for brain tumor identification beyond contrast enhancement on conventional images. However, clinical implementation is limited by long acquisition times, changing conditions between imaging and surgery, and lack of correlation with standard techniques.

**Purpose:**

To extend a methodology for multimodal data analysis to relaxometry data. To integrate relaxometry into the burr hole needle biopsy procedure with optical guidance, setup a workflow for multimodal data processing and analysis, and apply the methodology in a clinical setting.

**Methods:**

Multi-dimensional multi-echo relaxometry data (2x6 min) was acquired in addition to the clinical imaging protocol. Relaxation rate and proton density maps, as well as their differences were calculated before (R_1_, R_2_) and after gadolinium contrast-agent administration (R_1_Gd, R_2_Gd). Radiological volumes of interest (VOIs: tumor, edema, white matter, and biopsy) were defined on clinical images. Rate distribution changes were analyzed on three levels: the biopsied volume, along the needle trajectory (4x4x4 mm^3^ volumes), and VOIs. Increased R_1_Gd and R_2_Gd were compared to indications from 5-aminolevulinic acid-induced fluorescence and detailed neuropathological evaluation.

**Results:**

Neuropathological analysis confirmed seven glioblastoma, one lymphoma, and one non-tumorous diagnosis. Increased R_1_Gd was found in all biopsied volumes, although tumorous volumes presented larger R_1_Gd increase (3–9 times) compared to volumes dominated by necrotic or non-tumorous tissue. Along the trajectory, increased R_1_Gd and R_2_Gd were not tumor-specific, however, the greatest R_1_Gd shifts were found in or adjacent to radiologically defined tumorous tissue. Increased relaxation rates corresponded to 82% and 45% (R_1_Gd: φ = 0.35, R_2_Gd: φ = 0.27) of fluorescence peaks. In the radiological VOIs, increased R_1_Gd and R_2_Gd were found in tumorous tissue, a slight right shift in edematous tissue, and negligible changes in white matter.

**Conclusion:**

Combined analysis suggests increased R_1_Gd together with fluorescence peaks as a marker for tumor tissue. The presented multimodal approach provides a workflow toward clinical translation of relaxometry.

## Introduction

Brain tumor diagnoses rely on representative tissue samples taken during, e.g., a needle biopsy procedure. Due to the heterogeneous nature of high-grade brain tumors, finding representative samples is a challenging task [1,2].

Imaging techniques such as quantitative MRI (qMRI) hold potential for non-invasive tissue characterization by relating the measured signal to underlying physical properties [3,4] represented by quantitative maps. Relaxation rate maps are derived from a signal fit over multiple echo and delay times [5], and depict e.g., relaxation time (T) or rate (R = 1/T). Even though relaxometry was studied already in the 1970s, advancements in hardware and computing power have renewed its potential, indicating pathology in multiple sclerosis, epilepsy [6], and brain tumors. In two recent papers, a difference in T or R relative to administration of gadolinium-based (Gd) contrast media has been suggested as support for brain tumor tissue identification and infiltration beyond contrast enhancement on conventional MRI [7,8]. Blystad and colleagues assessed longitudinal relaxation rate (R_1_) difference before and after Gd contrast administration and demonstrated enhancement in the peritumoral zone in glioma patients [7]. In addition, Maurer et al. found a shortened longitudinal relaxation time (T_1_) after contrast in tumorous tissue for patients with isocitrate dehydrogenase (IDH) wildtype gliomas [8]. Although promising, extensive acquisition times have hampered the clinical pertinence of relaxometry. Moreover, changes between image acquisition and surgery limit the reliability of correlation studies between relaxometry data and standard techniques. These changes include patient positioning, registration errors during neuronavigation, movement as equipment is fastened, and altered intracranial conditions as the skull and dura are opened.

To overcome the limitations of preoperative imaging and identify tumorous tissue during surgery, intraoperative techniques have been introduced [9,10]. Previously, a system for optical intraoperative feedback of 5-ALA-induced tissue fluorescence through protoporphyrin IX (PpIX) accumulation associated with high-grade glioma and lymphoma [11,12] has been developed [13]. The system offers real-time measurements before tissue sampling through a probe-based system. Recently, the system was integrated into the surgical procedure [14] and pre-, intra-, and postoperative data combined for multimodal analysis. While intraoperative techniques allow feedback in situ, the possible adjustments are confined to the planned trajectory. Hence, there is still a need to improve representative tissue identification in target planning.

Therefore, the overall aim of this study was to extend the methodology for multimodal data analysis of pre-, intra-, and post-operative data to relaxometry on the millimeter scale. The specific aims were to integrate a relaxometry sequence suitable for clinical practice in the image acquisition protocol for brain tumor patients and set up a pipeline for registration to multimodal data, allowing comparison of relaxometry findings to fluorescence along the trajectory and neuropathological parameters in the biopsied volume. The methodology was applied in patients undergoing brain tumor needle biopsy surgery.

## Materials and methods

### Patients

Ten patients referred for brain tumor needle biopsy were included in the study (mean age 64 years, range 18–79 years, two women). The Ethical Review Authority granted study approval (2020–01404), and all patients gave written informed consent before inclusion (inclusion period: Jan 1^st^, 2022, to Aug 31^st^, 2023). In line with the clinical protocol for fluorescence-guided resection [12], a standard oral dose of Gliolan^®^ (20 mg/kg, Medac GmbH, Wedel, Germany) was given to each patient 2–3 hours before general anesthesia. One patient was excluded from analysis since the needle trajectory crossed the tentorium cerebelli, thus probing tissue outside the scope of relaxometry analysis. The optical data have previously been reported in a methodology study on the workflow for optical integration [14] and a 20-patient study of optical trends [15].

### Data acquisition

**Fig 1:**
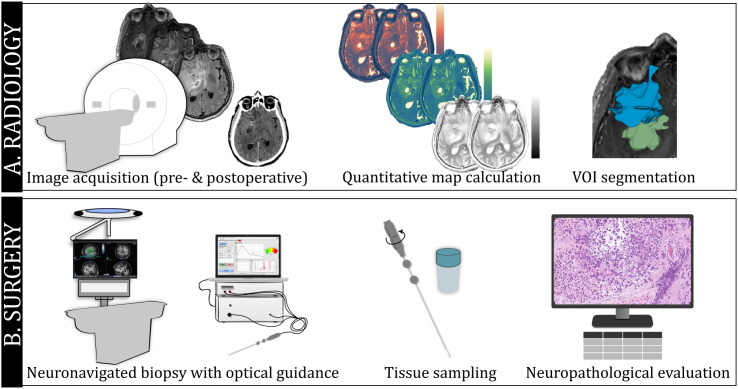
Overview of data acquisition with example data. (A) Radiology: clinical image acquisition (T_1_w gadolinium (Gd) contrast-enhanced and T_2_wFLAIR), calculated quantitative maps (R_1_, R_2_, PD before and after Gd contrast-enhancement), and example segmentation of radiological volumes of interest (VOI). (B) Surgical set up with neuronavigation and optical guidance, followed by tissue sampling and neuropathological evaluation of a Hematoxylin and Eosin-stained slide.

The multimodal data acquisition, including preoperative conventional imaging, relaxation rate map derivation, intraoperative optical measurements, and neuropathological diagnosis, is depicted in Fig 1.

### Radiology

MRI was acquired on a 3T scanner (Skyra or Prisma, depending on availability, Siemens Healthineers, Erlangen, Germany) within four days before surgery using a 20-channel head coil. The clinical brain tumor imaging protocol included conventional T_1_-weighted (w), T_2_w, T_2_w-FLAIR, and T_1_wGd contrast-enhanced images (Gd dose: 16–17 mL, Dotarem, 279 mg/mL, Gothia Medical, Sweden). A qMRI multi-dynamic multi-echo (MDME) sequence [5] was added before and after Gd administration, allowing calculation of several image contrasts from a single scan, see acquisition details in Table 1. Within 24 hours of surgery, a postoperative computed tomography (CT, SOMATOM Definition Edge, Siemens) or T_1_w MRI (3T Skyra, Siemens; 3T Ingenia or 1.5T Achieva, Philips, Best, The Netherlands; 1.5T Optima, GE Healthcare, Chicago, USA) was acquired.

**Table 1:** MRI acquisition parameters.

MRI sequence	2D/ 3D, Echo type	Reconstructed voxel size (mm^3^)	FOV (mm^2^)	TE (ms)	TR (ms)	TI (ms)	Acc type factor (ref lines)	Acquisition relative to Gd admin	Acquisition time (min:sec)
*T*_*1*_ *w*	3D GRE	1.0 x 1.0 x 1.0	256 x 176	2.26	2300	900	GRAPPA 2 (24)	Before, After	5:20
*T*_*2*_ *w*	3D SE	0.9 x 0.9 x 1.0	256 x 176	407	3200	–	GRAPPA 2 (24)	Before	4:50
*T*_*2*_ *w FLAIR*	3D SE	1.0 x 1.0 x 1.0	256 x 176	388	5000	1600	GRAPPA 3 (24)	Before	4:50
*qMRI MDME*	2D FSE	0.7 x 0.7 x 4.0, slice gap 1 mm	230 x 187	23, 106	4500	25	GRAPPA 3 (26)	Before, After^a^	6:00

Acc: acceleration, admin: administration, FLAIR: fluid-attenuated inversion recovery, FSE: fast spin echo, FOV: field of view, Gd: gadolinium, GRE: gradient echo, MDME: multi-dynamic multi-echo, qMRI: quantitative magnetic resonance imaging, ref: reference, SE: spin echo, TE: echo time, TI: inversion time, TR: repetition time, w: weighted.

^a^qMRIGd acquired after T_2_w, before T_1_wGd imaging.

Synthesized conventional images (synT_1_w, synT_2_w, synT_1_wGd, synT_2_wGd), relaxation time, and proton density maps (T_1_, T_1_Gd, T_2_, T_2_Gd, PD) were derived from the qMRI MDME sequence through the SyMRI software (v0.45.38, SyntheticMR AB, Linköping, Sweden) and relaxation rate maps (R_1_, R_1_Gd, R_2_, R_2_Gd) were calculated.

Radiological volumes of interest (VOIs) were defined by a senior neuroradiologist (I.B.) on preoperative conventional MR images in 3D Slicer [16]. Identified volumes were gross tumor volume (GTV) and peritumoral edema (PTE) in relation to the biopsy needle trajectory. The GTV was specified as the contrast-enhancing tumor on T_1_wGd, including the necrotic core and tumorous tissue in the T_2_w volume. PTE was identified as the hyperintense region on T_2_wFLAIR surrounding the GTV. Reference volumes of contralateral normal-appearing white matter (NAWM) VOIs (∅ 1 cm) were defined in axial slices on par with the lateral ventricle in the frontal and parietal lobes. In two patients, bilateral ischemic changes were present, thus, the NAWM VOI diameters were reduced (∅ 0.5 cm).

### Surgery

An in-house developed probe-based spectroscopy system [14,17] was used for intraoperative measurements of tissue fluorescence. In brief, tissue is excited by a near-UV laser, and three consecutive spectra within the wavelength (λ) interval 450–750 nm are captured and presented in real-time. The spectrum contains autofluorescence and, in the case of protoporphyrin IX (PpIX) accumulation, a peak at λ = 635 nm. Measurements were integrated into the surgical procedure by fitting the optical probe into the outer cannula of a biopsy needle (sampling window: 8 mm, Passive Biopsy Needle Kit, Medtronic Inc., MN, USA). In turn, the outer cannula was modified with an aperture at the tip to allow forward-looking measurements.

The needle biopsy procedure with optical guidance is described in detail in [14]. Briefly, a trajectory and target (defined as 0 mm) were planned on preoperative T_1_wGd or T_2_wFLAIR images. Images were registered to the patient’s anatomy with a neuronavigation system (StealthStation S8, Medtronic Inc.), and errors (registration and target) were noted. The skull and dura were opened (3.2–10 mm), and the AutoGuide^®^ (Medtronic Inc.) held the probe-cannula kit in place during manual insertion toward the target (step size: 1–13 millimeters). MRI coordinates and tissue fluorescence spectra were acquired in each position along the trajectory. The highest PpIX peak near the planned target was identified, and the biopsy cannula window was adjusted to overlap the specified area. Keeping the outer cannula in place, the probe was retracted, the inner cannula inserted, and tissue samples were taken in four directions, separated by a 90-degree needle rotation in the plane orthogonal to the trajectory. If the surgeon deemed the tissue material too sparse, another set of rotations or a second position was sampled. Tissue samples were sent for intraoperative smear analysis. After receiving a preliminary diagnosis from the neuropathologist, surgery was closed. Final diagnoses were based on the 2021 WHO classification of CNS tumors [18].

The formalin-fixed material was divided into 3 μm sections and stained with Hematoxylin and Eosin (H&E) and Ki67. Molecular analyses included O^6^-methylguanine-DNA-methyltransferase (MGMT) methylation (Therascreen MGMT Pyro kit, QIAGEN, Germany), IDH mutation (Therascreen IDH1/2 RGQ PCR kit, QIAGEN), and 1p19q codeletion. In addition to the clinical diagnosis, a senior neuropathologist (M.H.) re-evaluated the digitized stained slides. The re-evaluation assessed the morphological heterogeneity of the samples; presence and percentage of high- and low-grade tumor cells, non-tumor tissue, and necrosis.

### Data processing

The processing pipeline was built in Python (v.3.11) utilizing the Nipype framework [19] including image corrections with FMRIB’s Software Library [20] and co-registration with Advanced Normalization Tools (ANTs [21]). Detailed steps of the processing pipeline are illustrated in S1 Fig in the Supplemental Material.

Following bias field correction and skull-stripping, the conventional images were co-registered to T_1_wGd space using a rigid (preoperative) or affine (postoperative) transform. Additionally, the transform between the navigation system and native T_1_wGd space was calculated. A rigid transform was calculated between synT_2_w and synT_2_wGd space, followed by transform calculation between synT_1_wGd and T_1_wGd space (ANTs). GTV, PTE, and NAWM VOIs were transformed into native qMRI spaces for analysis.

The final entry and biopsy positions were identified on postoperative imaging. The entry point was defined by the trephination made in the skull (on CT) or opening in the dura (on MRI). The hypointense visible impact from trajectory and tissue sampling in the postoperative image was used to define the final biopsy position and volume. Measurement coordinates from the navigation system were aligned to the final trajectory by Euclidean distance. A volume (V_Traj_, 4x4x4 mm^3^; 25–37 voxels) was defined in each measurement position along the final trajectory. V_Traj_ and biopsy volumes were confirmed and classified according to tissue type (GTV, PTE, gray matter (GM), or WM) on conventional images by a senior neuroradiologist (I.B.). Volumes containing several tissue types, e.g., PTE and GTV, or GTV and WM, were identified.

Fluorescence signals were represented as a ratio between the maximum local intensity in the 633–637 nm range (corrected for autofluorescence contribution) and the maximum autofluorescence intensity around 500 nm. After disregarding noise-dominated spectra (signal-to-noise ratio 5:1), a PpIX peak was defined as a ratio >0.1.

### Data analysis and statistics

The translational displacement between qMRI acquisitions before and after Gd administration was quantified through the L2 norm. The Euclidean distance (median, interquartile range (IQR)) between planned and final trajectory positions was compared to the registration and target errors reported in the navigation system. Subsequent multimodal analysis was divided into the biopsied tissue volume, trajectory, and radiological VOI findings.

In the final biopsy volume, R_1_, R_2_, R_1_Gd, and R_2_Gd distribution changes were assessed through a two-sided Mann-Whitney-U-test corrected for Type 1 error of multiple comparisons using False discovery rate (FDR) with the Benjamini-Hochberg method [22] (p-values <0.05 were considered significant). Rate increases were investigated in relation to PpIX peak presence, tumor percentage, and Ki67 index.

Along the trajectory, R_1_, R_2_, R_1_Gd, and R_2_Gd in each V_Traj_ were compared with a two-sided Mann-Whitney-U-test using FDR or multiple comparison correction (corrected p-value <0.05). Inter-modality correlations between R_1_, R_2_, R_1_Gd, and R_2_Gd V_Traj_ distributions, radiological classification on conventional imaging, and fluorescence were assessed via the Phi coefficient (φ). Per tissue type density heatmaps of R_1_, R_2_, R_1_Gd, and R_2_Gd were investigated and compared to the corresponding VOI values.

Finally, the median, IQR, unbiased skew, and voxel count for R_1_, R_2_, R_1_Gd, and R_2_Gd, and PD distributions in the radiological VOIs (GTV, PTE, NAWM) were reported.

## Results

A qMRI relaxometry protocol was integrated into clinical practice, adding 2x6 min to the imaging acquisition time. The translational displacement between pre- and postoperative imaging was 0.42–6.3 mm. The median Euclidean distance between the planned and final trajectory was 2.8 mm (1.6–7.0 mm) while the intraoperative registration and target errors in the navigation system were 1.5  mm (1.5-1.6 mm) and 0.4  mm (0.2-0.5 mm). Neuropathological diagnoses were Glioblastoma IDH-wildtype, CNS WHO grade 4 (Patients 1–7), Primary diffuse large B-cell Lymphoma (Patient 8), and non-tumor (Patient 9). A noticeable increase in astrocytes was found for the non-tumor sample, but no indication of malignancy.

### Biopsied tissue volume

Neuropathological data, relaxation rates, and PpIX peak occurrence are presented in Table 2. Overall, increased R_1_Gd was observed in seven tumorous and non-tumorous biopsy volumes, and R_2_Gd was increased in four tumorous samples. Relaxation rates showed a large variability within and between patients. PpIX peaks were found in tumorous biopsy volumes but not in the non-tumorous samples. The neuropathological re-evaluation revealed that two of the tumorous samples contained a majority of non-tumor tissue (Patient 1) or necrosis (Patient 7).

**Table 2:** Neuropathological diagnosis, tumor percentage, relaxation rates, and proton density values from the biopsied volume.

Patient [#]	Tissue sample diagnosis	CNS WHO 2021 grade	IDH mut/ wt	MGMT-meth	Ki67 index [%]	Tumor HG (LG) [%]	Marg. Zone [%]	Non-tumor [%]	Nec-rosis [%]	R_1_ [s^-1^]	p(ΔR_1_)	R_2_ [s^-1^]	p(ΔR_2_)	PD [%]	p(ΔPD)
1	Glioblastoma	4	wt	unmeth	15	40	5	55	0	0.26 (0.24-0.40)		3.3 (2.8-6.2)		107 (103-107)	n.s.
										0.83 (0.48-1.1)	< 0.001	5.3 (3.7-7.6)	0.003	106 (105-120)	(0.14)
2	Glioblastoma	4	wt	unmeth	38	95	0	0	5	0.69 (0.65-0.74)		10 (9.7-11)	n.s.	87 (85-89)	
										1.2 (1.1-1.3)	< 0.001	11 (9.7-11)	(0.13)	91 (85-98)	0.004
3	Glioblastoma	4	wt	unmeth	50	60	30	0	10	0.52 (0.49-0.56)		8.3 (7.8-8.9)		87 (83-90)	
										1.3 (0.68- 1.4)	< 0.001	9.4 (8.2-10)	<0.001	98 (85-98)	< 0.001
4	Glioblastoma	4	wt	meth	24	100	0	0	0	0.57 (0.55-0.63)		9.5 (9.2-9.9)		97 (95-99)	
										0.96 (0.90-1.0)	< 0.001	10 (10-11)	< 0.001	109 (104-111)	< 0.001
5	Glioblastoma	4	wt	meth	34	15	50	15	0	0.74 (0.71-0.85)		10 (9.5-11)		85 (81-89)	n.s.
						(20)				1.1 (0.99-1.2)	< 0.001	11 (10-12)	< 0.001	84 (81-88)	(0.25)
6	Glioblastoma	4	wt	unmeth	32	60	40	0	0	0.89 (0.85-0.95)		9.7 (8.8-11)		98 (90-103)	
										1.8 (1.4-2.0)	< 0.001	8.7 (7.4-9.6)	0.002	118 (114-126)	< 0.001
7	Glioblastoma	4	wt	unmeth	46	30	0	0	60	1.3 (1.2-1.4)		7.0 (6.8-7.5)	n.s.	109 (107-110)	
						(10)				1.3 (1.2-1.3)	0.03	7.4 (6.6-8.1)	(0.08)	102 (100-104)	< 0.001
8	Lymphoma	–	–	–	90	100	–	0	-	0.73 (0.64-0.88)		11 (8.9-11)	n.s.	91 (87-106)	
										1.4 (1.1-1.6)	< 0.001	11 (8.1- 12)	(0.08)	100 (95-111)	< 0.001
9	Non-tumor	–	–	–	–	–	–	100^a^	-	0.66 (0.62-0.71)		7.7 (6.9-8.6)	n.s.	91 (83-93)	
										0.76 (0.73-0.80)	< 0.001	7.8 (7.5-8.0)	(0.54)	82 (80-84)	< 0.001

Statistically significant differences before and after Gd (p<0.05) are highlighted in gray. Gd: gadolinium, GTV: gross tumor volume, HG: high-grade, LG: low-grade, IDH: isocitrate dehydrogenase, IQR: interquartile range, meth: methylated, MGMT: O6-methylguanine-DNA-methyltransferase, mut: mutated, n.s.: non-significant, PD: proton density, R_1_, R_2_: longitudinal and transverse relaxation rate, wt: wildtype.

^a^Increased level of astrocytes, no indication of malignancy.

**Fig 2:**
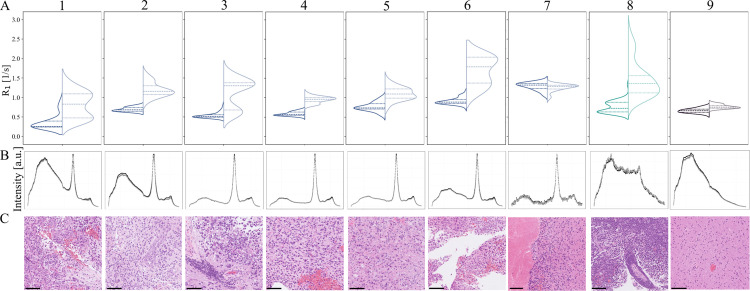
Longitudinal relaxation rates (R_1_), fluorescence response, and Hematoxylin & Eosin-stained (H&E) neuropathology slides from the biopsied volume. (A) R_1_ value distributions before (left violin) and after (right violin) gadolinium (Gd) administration per patient. Patients 1-7: glioblastoma (blue), Patient 8: lymphoma (turquoise), Patient 9: non-tumor (black). The dashed lines denote the population median, upper, and lower quartiles. (B) Fluorescence response with or without PpIX peak. (C) H&E-stained frozen section from digital pathology PACS. The black horizontal lines indicate 100 μm.

Joint assessment showed agreement between increased R_1_Gd, PpIX peaks, and tumor diagnoses in seven patients, while Patient 7 presented decreased R_1_Gd and PpIX peaks, see Fig 2. Tumorous biopsy volumes had a larger increase in R_1_Gd than necrotic or non-tumorous volumes (3–9 times). Increased R_2_Gd and PpIX peaks were found in the biopsy volume of four patients, and decreased R_2_Gd in one patient. Interestingly, PD percentages included values >100% and showed variable trends after Gd administration compared to before (Table 2: five increased, two unchanged, two decreased).

### Along the trajectory

**Fig 3:**
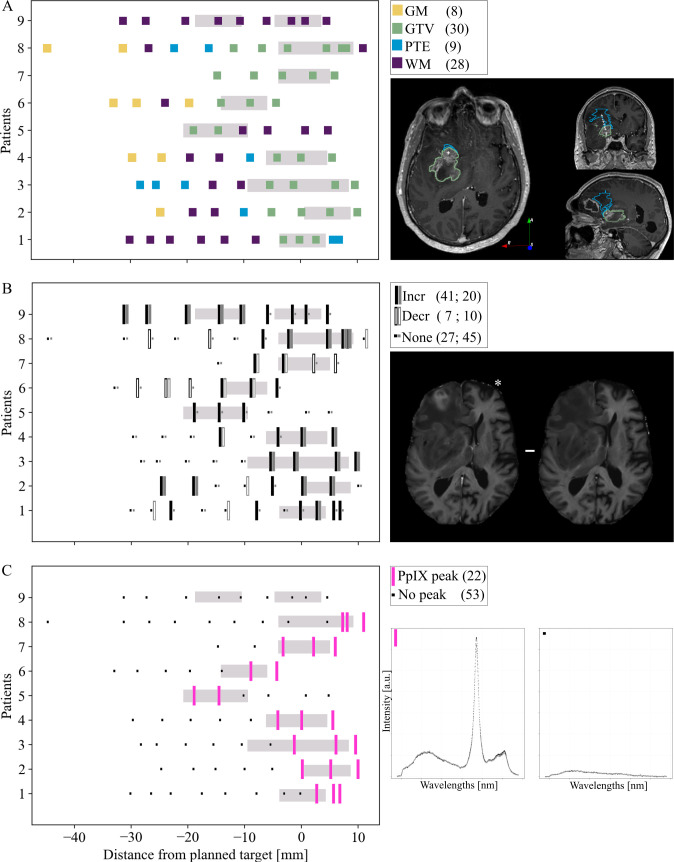
Multimodal trajectory data. Left column: Radiological classification, R_1_ relaxation rate difference, and fluorescence peaks in relation to distance from planned target position (0 mm). Right column: Data examples. A) Radiological classification of measurement volume into GM, GTV, PTE, or WM on clinical imaging. (B) Longitudinal (R_1_, black) and transverse (R_2_, gray) relaxation rates after Gd compared to before Gd contrast-agent administration (corrected p-value < 0.001). Filled lines indicate an increase, and hollowed-out lines indicate a decrease. C) PpIX-fluorescence peak positions, i.e., where fluorescence ratio >0.1. Gray boxes correspond to the needle biopsy volumes (8 mm) defined by the final biopsy position on postoperative imaging. The larger volumes for patients 3, 8, and 9 are due to the surgeon deeming the material at the first biopsy position too sparse. Values in parenthesis specifies the total number of volumes for that tissue and data type. Gd: gadolinium, GM: gray matter, GTV: gross tumor volume, PpIX: protoporphyrin IX, PTE: peritumoral edema, WM: white matter.

The multimodal needle trajectory data per patient are presented in Fig 3. Along the needle trajectory (V_Traj_ = 75), radiological definition of tumor (GTV) spanned −18 mm to +10 mm of the planned target (Fig 3A). Increased R_1_Gd was observed in all tissue types, along the whole trajectory (φ: 0.39, Fig 3B). R_2_Gd increases were found in all but PTE tissues, also covering the whole trajectory (φ: 0.31, Fig 3B). In agreement with radiological definition of GTV, PpIX fluorescence peaks ranged from −18 mm to +10 mm (φ: 0.61, Fig 3C).

Combined analysis suggested an overlap between PpIX peaks and increased R_1_Gd (φ: 0.35, 18/22 V_Traj_). These V_Traj_ corresponded to the radiological definition of GTV but also included PTE (Patient 1). The four V_Traj_ where PpIX fluorescence but no increased R_1_Gd was found were classified as GTV and WM on conventional imaging (Patients 2, 7 and 8). Four GTV V_Traj_ did not exhibit any R_1_Gd increase or PpIX fluorescence (Patients 1, 7 and 8). The overlap between PpIX peaks and increased R_2_Gd was lower than that of R_1_Gd and confined to V_Traj_ defined as GTV (φ: 0.27, 10/22 V_Traj_). Per patient, per V_Traj_ rate distributions together with the fluorescence ratio are presented in S2 and S3 Figs in the Supplemental Material.

**Fig 4:**
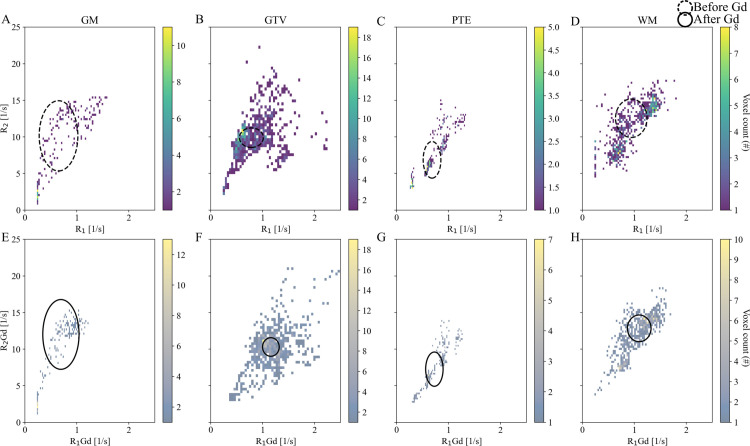
Relaxation rate (R_1_, R_2_) density heatmaps of trajectory data. (A-D) Per tissue distribution before and (E-H) after gadolinium (Gd) contrast enhancement. (A, E) Gray matter (GM). (B, F) Gross tumor volume (GTV). (C, G) Peritumoral edema (PTE), and (D, H) White matter (WM). Patient 9 had no radiological definition of GTV or PTE. Circles depict median and IQR before (dotted line) and after administration of Gd (solid line).

GTV V_Traj_ showed greater R_1_ and R_2_ value dispersion than the other tissue types (Fig 4A-D), ranging from the cerebrospinal fluid-to-GM axis (values similar to PTE) to values greater than that of WM. Additionally, a trend of greater R_1_Gd shifts in GTV V_Traj_ than GM, PTE, and WM was observed (Fig 4E-H, Table 3).

**Table 3:** Relaxation rates, proton density, and fluorescence values per radiological tissue classification on conventional imaging along the trajectory.

Manual radiological classification	V_Traj_ [n]	R_1_ [s^-1^]		R_2_ [s^-1^]		PD [%]		Fluorescence ratio [a.u.]
		**Median (IQR)**	**p(ΔR**_**1**_)	**Median (IQR)**	**p(ΔR**_**2**_)	**Median (IQR)**	**p(ΔPD)**	**Median (IQR)**
GM	8	0.65 (0.27-1.0)	n.s.	10 (3.5-13)	n.s.	89 (68-105)	n.s.	0.0 (0.0-0.0)
		0.69 (0.23-0.93)	(0.27)	12 (3.7-13)	(0.30)	90 (76-106)	(0.18)	
GTV	30	0.78 (0.60-1.1)		9.9 (8.6-11)		89 (81-98)		1.0 (0.0-2.9)
		1.2 (1.0-1.3)	<0.001	10 (9.0-11)	0.003	97 (88-104)	<0.001	
PTE	9	0.68 (0.57-0.91)	n.s.	6.9 (5.3-10)	n.s.	83 (77-87)	n.s.	0.0 (0.0-0.0)
		0.73 (0.58-0.92)	(0.55)	7.3 (5.4-10)	(0.45)	83 (75-90)	(0.99)	
WM	28	0.94 (0.71-1.3)		13 (8.8-14)		69 (63-81)		0.0 (0.0-0.0)
		1.1 (0.83-1.3)	<0.001	13 (10-14)	0.04	68 (62-78)	0.03	

Statistically significant differences before and after Gd (p < 0.05) are highlighted in gray. Gd: gadolinium, GM: gray matter, GTV: gross tumor volume, IQR: interquartile range, n.s.: non-significant, p: p-value, PD: proton density, PTE: peritumoral edema, R_1_, R_2_: longitudinal and transverse relaxation rate, WM: white matter.

### Radiological volumes of interest

**Fig 5:**
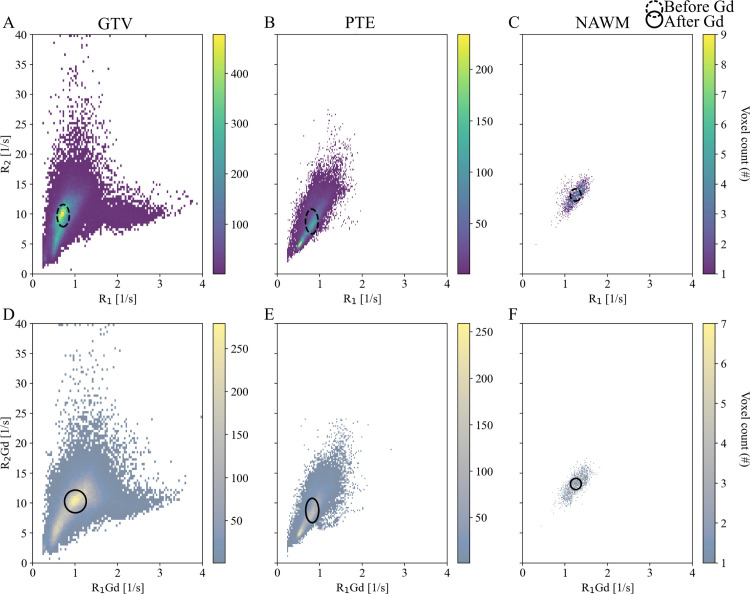
Relaxation rate (R_1_, R_2_) density heatmaps of radiological volumes of interest. (A-C) Per tissue distribution before and (D-F) after gadolinium (Gd) contrast enhancement. (A, D) Gross tumor volume (GTV). (B, F) Peritumoral edema (PTE), and (C, F) Normal appearing white matter (NAWM). Patient 9 had no radiological definition of GTV or PTE. Circles depict median and IQR before (dotted line) and after administration of Gd (solid line).

General trends of lower R_1_ and R_2_ in GTV compared to NAWM were indicated in the density heatmaps (Fig 5, Table 4). Additionally, increased R_1_Gd was observed in the GTV, a slight right shift in the PTE VOI, and constant values in the NAWM VOI (Table 4).

**Table 4:** Relaxation rate and proton density in radiological VOIs before and after Gd administration.

Manual radiological classification	R_1_ [s^-1^]		R_2_ [s^-1^]		PD [%]		Voxel Count
	**Median (IQR)**	**Skewness**	**Median (IQR)**	**Skewness**	**Median (IQR)**	**Skewness**	
**GTV**	0.72 (0.60-0.89)	2.7	9.7 (7.7-11)	0.94	87 (79-95)	−0.07	60 x 10^3^
	1.0 (0.75-1.3)	0.98	10 (8.1-12)	0.72	88 (81-96)	0.08	
**PTE**	0.81 (0.65-0.95)	0.51	8.8 (6.9-11)	0.66	79 (74-86)	−0.06	36 x 10^3^
	0.83 (0.66-0.97)	0.68	8.8 (6.9-11)	0.65	79 (73-85)	−0.14	
**NAWM**	1.3 (1.1-1.4)	−0.34	13 (12-14)	−0.30	64 (62-68)	0.85	1.9 x 10^3^
	1.3 (1.1-1.4)	−0.16	13 (12-14)	−0.21	64 (60-67)	0.43	

Gd: gadolinium, GTV: gross tumor volume, IQR: interquartile range, NAWM: normal appearing white matter, PD: proton density, PTE: peritumoral edema, R_1_, R_2_: longitudinal and transverse relaxation rate, VOI: volume of interest.

Compared to the relaxation rates in the VOIs, median R_1_ along the trajectory was higher for GTV and lower for PTE and WM. Median R_2_ was skewed toward higher values for GTV and lower for PTE. For WM, the values were comparable. An increase in PDGd percentage was found for GTV. Median R_1_ rates in the biopsied volumes were lower than those of NAWM for eight patients and similar to NAWM for one patient (Patient 7).

## Discussion

Reliable identification of representative tissue for tumor diagnosis would be exceptionally valuable for the clinical treatment of brain tumors. The present study added qMRI relaxometry with clinically acceptable acquisition times to the routine imaging protocol and a processing pipeline for multimodal brain tumor biopsy data analysis. Multimodal analysis indicated that increased R_1_Gd and R_2_Gd values were not tumor-specific, but R_1_Gd increase was greater in tumorous tissue compared to non-tumorous or necrotic tissue, as confirmed by neuropathology. Increased R_1_Gd was also found in 82% of PpIX peak positions. To our knowledge, this is the first study to compare relaxometry to multimodal pre-, intra-, and post-operative data on the millimeter scale.

### Relaxation rates after gadolinium contrast enhancement

#### In the gross tumor volume.

A general trend of increased R_1_Gd (T_1_ shortening) was found in tissue radiologically defined as GTV, as corroborated by other studies [7,8,23,24]. The increase indicates interaction of Gd with tissue outside vessels, signifying a leaky blood-brain barrier [8,25]. The relaxation rate increase is proportional to tissue Gd concentration [7,26]. Although longitudinal relaxation effects (R_1_) of Gd tend to dominate [27], a slight right shift in R_2_Gd was seen in the radiological GTV VOI, along the trajectory in V_Traj_ classified as GTV and WM, and in five biopsy volumes.

#### Beyond the gross tumor volume.

Outside the radiologically defined tumor volume, a slight right shift in R_1_Gd was found for the PTE VOI. Blystad and colleagues presented increased R_1_Gd in PTE close to GTV, indicating enhancement beyond what is seen on conventional imaging [7]. In the present study, four WM and PTE dominant V_Traj_ with increased R_1_Gd were found close to the radiological definition of GTV. Interestingly, four V_Traj_ close to GTV showed decreased R_1_Gd (GTV, PTE, and GM). The altered rates could be due to edema, noise, or potentially, partial volume effects arising from the 2D acquisition technique.

Another thirteen volumes (GM: n = 1, WM: n = 12), including all V_Traj_ for Patient 9, showed increased R_1_Gd (Fig 3B). Notably, for Patient 9, R_1_ was lower than otherwise found in WM, no PpIX fluorescence was found, and the neuropathological analysis of the two biopsy samples showed an increase of astrocytes but no sign of malignancy. Other studies have found small relative increases after Gd in non-tumorous tissue, e.g., in contralateral control tissue [24], offering a potential explanation for the observed R_1_Gd increase in Patient 9. Another potential influence could be movement during image acquisition, especially along the z-direction.

Notably, R_1_ for Patient 7 was comparable to that of NAWM rather than GTV, as indicated by the radiological VOI analysis , and a slight decrease in R_1_Gd was observed (Fig 3B). The restricted response could be due to preoperative bleeding, or the majority of necrotic tissue (60%) identified in the neuropathological re-evaluation.

These results support the finding that R_1_Gd increase is not tumor-specific and could benefit from combined analysis with other measures.

### Increased R_1_Gd rates overlap fluorescence peaks and tumor percentage

Over 80% of the PpIX peaks corresponded to an increase in R_1_Gd. Interestingly, the PpIX response seems delayed compared to the increase in R_1_Gd, see Patients 2, 7, and 8 in Figs 3B and 3C. This could be explained by the volume differences between fluorescence penetration depth (<1mm) and the slice thickness of the qMRI sequence (4 mm). Similar to the R_1_Gd increase, PpIX peaks were found in PTE (n = 2) and WM (n = 1) beyond the radiological definition of GTV. PpIX accumulates intracellularly in neoplastic tissue in proximity to a leaky blood-brain barrier. The mechanisms behind tumor selectivity for PpIX accumulation are not fully known but have been attributed to several cellular factors, including altered pH and enzyme concentrations [28].

Tumor tissue heterogeneity is particularly evident in Patients 1, 5, and 7, where the biopsy sample included viable tumor, necrotic, and non-tumor tissue. These heterogeneities result in altered relaxation rates and fluorescence responses compared to if only viable tumor tissue were present. However, to diagnose a glioblastoma, necrosis or microvascular proliferant vessels need to be present in the sample, or since 2021, certain molecular criteria are also accepted [18].

As Novikov et al argue [29], MRI and neuropathology have limited comparative value since the techniques are not sensitive to the same parameters. However, as neuropathology is the current diagnostic tool for tumors, the comparison is not only needed but necessary for exploring the potential of qMRI in clinical applications.

### Relaxation rates and proton densities across tissue types

In the radiological VOIs, decreased R_1_ in GTV and PTE compared to NAWM were identified, analogous to the prolonged T_1_ times previously presented [8]. T_1_ lengthens (i.e., R_1_ decreases) as the amount of free water increases, e.g., in PTE compared to WM [3] or in necrotic tissue [7]. R_1_ also decreases with intracellular water content and increases with the concentration of macromolecules and iron [30,31]. The relaxation rates for NAWM in this study are comparable to those reported in the literature [7,32]. Likewise, the decreased R_2_ in GTV was in line with previous findings [8]. R_2_ is receptive to dipole interactions with the surrounding tissue [3], decreases with the fraction of free water, and increases with iron content [8].

In normal-appearing brain tissue, Hagiwara and colleagues found a lower PD in WM compared to GM [32], which is corroborated by the present study. Proton density percentages >100 were found in manual radiological VOIs (GTV, PTE), most likely due to their edematous nature. As PD is inversely dependent on the macromolecule concentration, an increase in PD indicates increased free water content in the voxel [31]. Altered PDGd percentages were observed in the GTV VOI, V_Traj_ GTV, and seven biopsy volumes. As no PD percentage change between acquisitions is expected, this could be an effect of partial volumes or the PD calculations. As PD is calculated based on parameters from the other maps, including flip and saturation angle, repetition and echo times, T_1_ and T_2_ times, and an intensity scaling factor [32], any uncertainties in the previous measurements will be amplified in PD.

### Postoperative imaging

In the present study, the registration errors reported in the navigation system were smaller than the calculated Euclidean distances between the pre- and postoperatively defined trajectories. Although the Euclidean distance is a compound measure, including the registration error during surgery, movement during equipment fastening, potential brain shift, and errors from image co-registration, the discrepancy between the pre- and postoperative trajectory is prominent. As postoperative image is not a part of the clinical routine for needle biopsy patients, and correlation studies often performed on preoperative data, we encourage caution. Especially when imaging at higher spatial resolution, postoperative imaging, and a robust image co-registration pipeline are necessities for accurate interpretation.

### Limitations

A 2D sequence with a slice thickness of 4 mm was used for qMRI acquisition in the present study, introducing inevitable partial volume effects (PVE). These effects are further accentuated if the patient has moved along the z-axis. More specifically, major (n = 3), minor (n = 3), and negligible movement (n = 3) were observed in this cohort. The largest translational displacement between qMRI acquisitions was found for Patient 9. In future studies, the use of a 3D sequence is highly encouraged to reduce PVE. In the context of the 2D sequence, attempts were made to minimize the influence of PVE by performing the analysis in the native R_1_, R_2_, R_1_Gd, and R_2_Gd spaces. Consequently, no voxel-to-voxel differences were calculated, but rather the distribution changes over each volume (biopsy, V_Traj_, and VOIs) were analyzed.

It should be noted that the radiological VOIs (GTV, PTE, and NAWM) contain a large number of voxels (2x10^3^ - 64x10^3^). Thus, the statistical differences in qMRI values were not reported for these volumes. The number of measurements in the V_Traj_ radiological classes was also unbalanced, varying from 8 (GM) to 30 (GTV) measurements. Another limitation was the small size and heterogeneity of the patient cohort, requiring further data collection to confirm the findings of the present study.

Herein, measurements on several different scales are compared, ranging from micrometer sections in the neuropathological analysis to several millimeters for the surgical procedure. The results were reported on the millimeter scale, as this is the scale of significance during surgery.

## Conclusions

A methodology for multimodal pre-, intra-, and postoperative data combining radiology, PpIX-fluorescence, and neuropathology on the millimeter scale was extended to qMRI relaxometry. The combined analysis suggests increased R_1_Gd and fluorescence peaks as a marker for high-grade tumor tissue, such as glioblastoma and lymphoma, confirmed by neuropathology. Relaxometry could offer further insights into tissue characteristics beyond contrast enhancement on conventional MRI, however, its implications should be interpreted in conjunction with other modalities, e.g., PpIX-fluorescence and neuropathology, until its mechanisms are fully elucidated.

## Supporting information

S1 Fig Detailed image processing pipeline of preoperative clinical and quantitative MRI as well as postoperative MRI or CT imaging. T_1_wGd space is used as reference space unless otherwise stated. ANTs: advanced normalization tools, FOV: field of view, FSL: FMRIB’s Software Library, GD: gadolinium, GM: gray matter, GTV: gross tumor volume, NAWM: normal appearing white matter, PTE: peritumoral edema, syn: synthetic, VOI: volume of interest, w: weighted.
(DOCX)

S2 Fig Longitudinal relaxation rate (R_1_) and fluorescence ratio in V_Traj_ along the biopsy trajectories. Violin plots represent R_1_ distribution before (left) and after (right) gadolinium administration, crosses depict the fluorescence ratio, circles signify conventional radiological classification, and gray blocks specify biopsy sampling volume. GM: gray matter, GTV: gross tumor volume, Pat: Patient, PTE: peritumoral edema, WM: white matter.
(DOCX)

S3 Fig Transverse relaxation rate (R_2_) and fluorescence ratio in VTraj along the biopsy trajectories. Violin plots represent R_2_ distribution before (left) and after (right) gadolinium administration, crosses depict the fluorescence ratio, circles signify conventional radiological classification, and gray blocks specify biopsy sampling volume. GM: gray matter, GTV: gross tumor volume, Pat: Patient, PTE: peritumoral edema, WM: white matter.
(DOCX)
