# Supplementary material for: Quantitative MRI relaxometry in brain tumor needle biopsies: Multimodal comparison with tissue fluorescence, radiology, and neuropathology
Source: PLoS One. 2025 Jul 7;20(7):e0326765. doi: 10.1371/journal.pone.0326765 (PMC12233892; doi:10.1371/journal.pone.0326765)
Supplement: S1 Fig — T1wGd space is used as reference space unless otherwise stated. ANTs: advanced normalization tools, FOV: field of view, FSL: FMRIB’s Software Library, GD: gadolinium, GM: gray matter, GTV: gross tumor volume, NAWM: normal appearing white matter, PTE: peritumoral edema, syn: synthetic, VOI: volume of interest, w: weighted. (DOCX) [file pone.0326765.s001.docx]

**
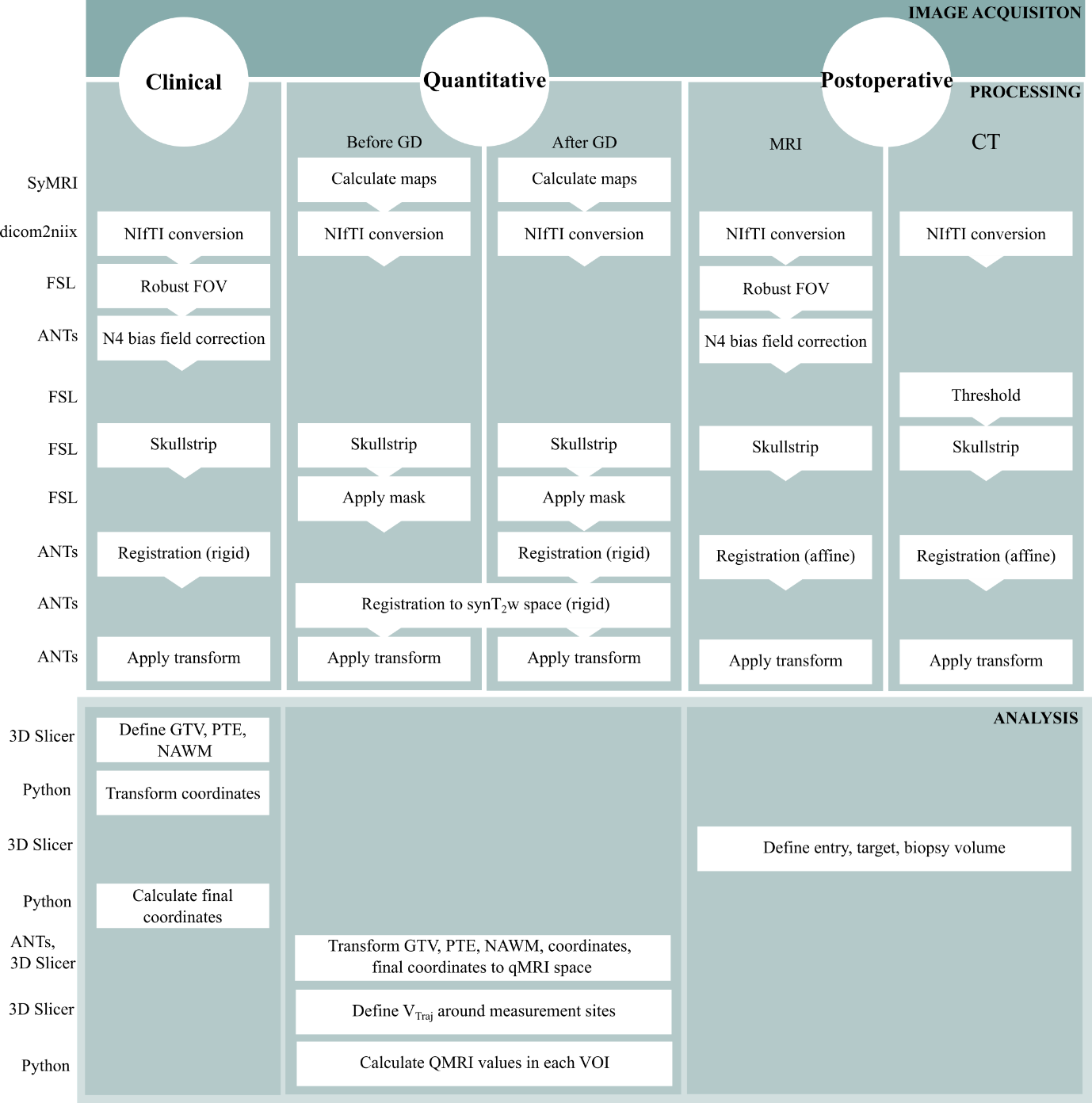
**

**S1 Fig. Detailed image processing pipeline of preoperative clinical and quantitative MRI as well as postoperative MRI or CT imaging.** T_1_wGd space is used as reference space unless otherwise stated. ANTs: advanced normalization tools, FOV: field of view, FSL: FMRIB’s Software Library, GD: gadolinium, GM: gray matter, GTV: gross tumor volume, NAWM: normal appearing white matter, PTE: peritumoral edema, syn: synthetic, VOI: volume of interest, w: weighted
