# Supplementary material for: Quantitative MRI relaxometry in brain tumor needle biopsies: Multimodal comparison with tissue fluorescence, radiology, and neuropathology
Source: PLoS One. 2025 Jul 7;20(7):e0326765. doi: 10.1371/journal.pone.0326765 (PMC12233892; doi:10.1371/journal.pone.0326765)
Supplement: S3 Fig — Violin plots represent R2 distribution before (left) and after (right) gadolinium administration, crosses depict the fluorescence ratio, circles signify conventional radiological classification, and gray blocks specify biopsy sampling volume. GM: gray matter, GTV: gross tumor volume, Pat: Patient, PTE: peritumoral edema, WM: white matter. (DOCX) [file pone.0326765.s003.docx]

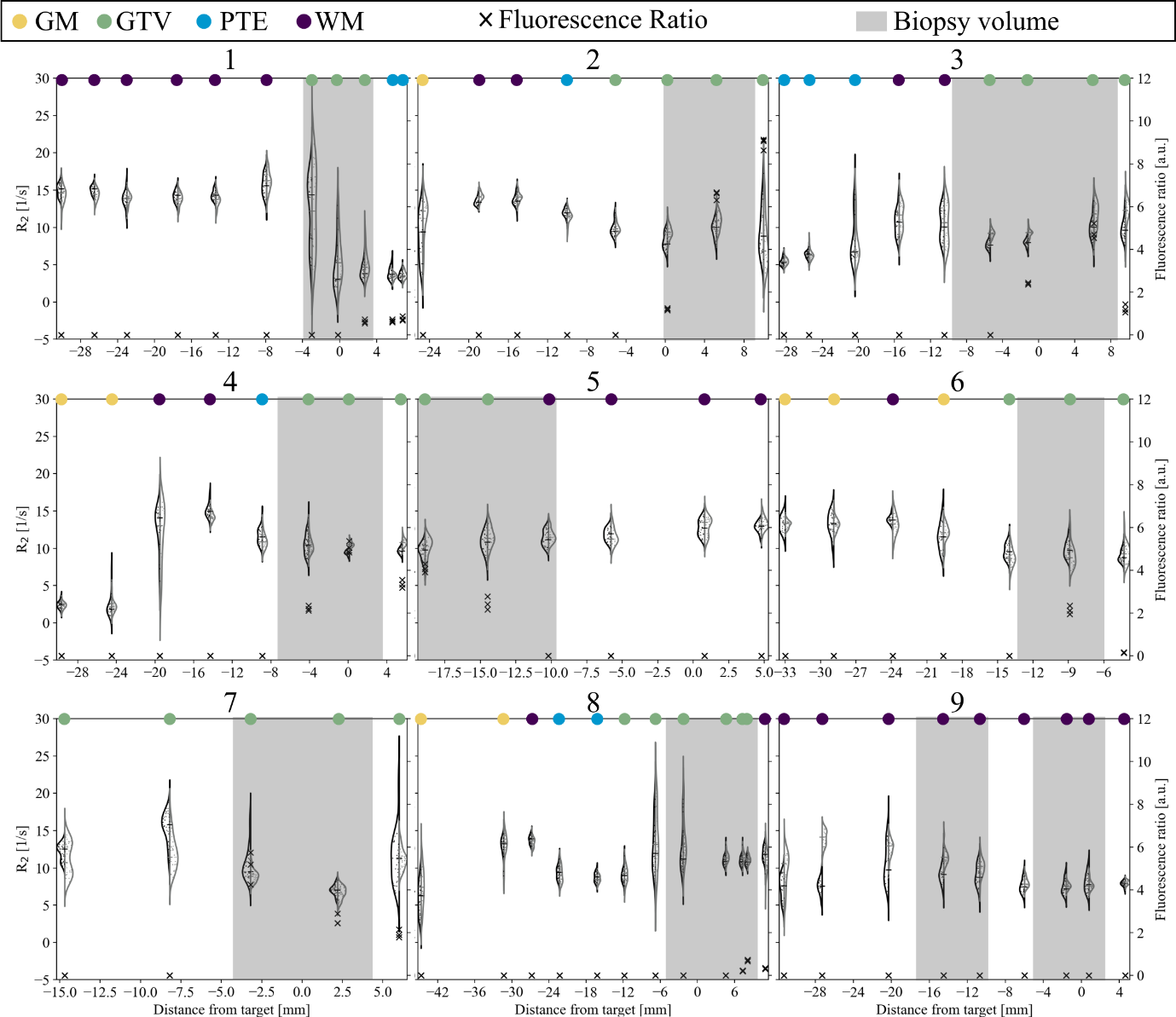


**S3 Fig. Transverse relaxation rate (R_2_) and fluorescence ratio in V_Traj_ along the biopsy trajectories.** Violin plots represent R_2_ distribution before (left) and after (right) gadolinium administration, crosses depict the fluorescence ratio, circles signify conventional radiological classification, and gray blocks specify biopsy sampling volume. GM: gray matter, GTV: gross tumor volume, Pat: Patient, PTE: peritumoral edema, WM: white matter
